# Decreasing Prescriber Errors in a Tertiary Care Pediatric Intensive Care Unit via QI Methodology

**DOI:** 10.1097/pq9.0000000000000909

**Published:** 2026-09-22

**Authors:** Timothy Brandt, Dhara D. Shah, Gina Cassel-Choudhury

**Affiliations:** From The Children’s Hospital at Montefiore, Bronx, N.Y.

## Abstract

**Introduction:**

Medication errors are a significant source of preventable adverse drug events for pediatric patients, particularly those with a complex chronic condition (CCC). In our pediatric intensive care unit (PICU), medication errors often occurred during admission or as medication regimens were adjusted throughout hospitalization. Our primary aim was to reduce the rate of reported prescriber medication errors per 1,000 medication orders by 40% between August 2022 and June 2024.

**Methods:**

Key drivers contributing to prescriber medication errors included the lack of a standardized medication reconciliation process, high-risk medications unintentionally expiring, and the difficulty of reconciling the home medication list for patients with a CCC. Five plan-do-study-act interventions were completed. A retrospective chart review was used for all baseline data. The primary outcome was the rate of reported monthly prescriber medication errors per 1,000 medication orders placed. This project took place in a 26-bed PICU within an urban, tertiary care children’s hospital.

**Results:**

The rate of reported prescriber medication errors remained stable throughout the intervention period without evidence of special cause variation. However, admission medication reconciliation errors among children with CCCs decreased by a mean of 43% over time, with a downward centerline shift.

**Conclusions:**

A multidisciplinary quality improvement approach was associated with improvement in medication reconciliation processes for children with CCCs. Pharmacy-supported medication reconciliation and standardized workflows were associated with fewer admission medication reconciliation errors in this high-risk population.

## INTRODUCTION

Medication errors are a significant source of preventable adverse events, especially for pediatric patients. A large prospective observational study published in 2024 evaluated 5,137 medication administrations in hospitalized children and found that medication administration errors occurred in 37.0% of administrations, with 25.8% of those errors considered potentially serious.^1^ Medication errors are up to three times more likely to occur in pediatric patients^2–5^ due to factors such as the need for weight-based dosing, the limited availability of pediatric formulations, and the developmental limitations of pediatric patients.^6^ The admission process is a particularly noteworthy source of medication errors, with approximately 62% of errors reaching the patient.^7–11^

Efforts to minimize medication errors have been a national priority for several decades, including initiatives from the Institute for Healthcare Improvement and the Joint Commission’s national performance goals.^12,13^ The process of accurate medication reconciliation, especially when bundled with other interventions, has been shown to reduce medication errors, adverse drug events, and healthcare utilization.^14,15^ Studies have shown that pharmacy involvement is the gold standard for optimal medication reconciliation.^15^

Accurate medication reconciliation is especially important for children with a complex chronic condition (CCC), defined as a medical condition expected to last longer than 12 months and requiring subspecialist care. Patients with a CCC are a growing population in pediatric intensive care units (PICUs).^16,17^ Pediatric-onset chronic conditions have increased from 23% in 2000 to 30% in 2018, accounting for roughly 130,000 additional children with chronic conditions or functional limitations diagnosed annually.^18^ Patients in the PICU with CCC include those with epilepsy, medical technology dependence, or genetic syndromes. Studies demonstrate that these patients take an average of 5.3 chronic medications and that each extra medication increases their risk of potential adverse drug events, with 16% of these potential adverse drug events having the potential for serious harm.^7,8^

Our institution’s PICU did not have a standardized process for medication reconciliation on admission or daily rounds, and specific team members were not assigned to ensure the accurate completion of this task. Patients often had medication errors such as incorrect doses or inappropriate timing of medications, which could go uncorrected for several days. Medications would unintentionally expire or be completed in the electronic health record (EHR), leading to medication omissions. These problems were even more apparent for patients with CCC, who often have complex home medication regimens.

We defined prescriber medication errors as errors including incorrect medication selection, dose, route, frequency/timing, omission, and unintended medication discontinuation. Admission medication reconciliation errors represented a subset of prescriber medication errors involving discrepancies between the intended medication regimen, including home medications, and the active medication orders within the patient’s first 24 hours of admission.

### Rationale

Pediatric patients with a CCC have multiple opportunities for latent prescriber medication errors, which we wanted to address. Critically ill patients often require adjustments to their medications, leading to confusion as hospital providers rotate. This risk may be alleviated with frequent and standardized reviews of a patient’s intended medication list. Patients may have multiple subspecialty providers and inaccessible medical records. This can make the process of completing an accurate home medication reconciliation list time-consuming. Pharmacy assistance in completing complex home medication reconciliations may allow for more accurate home medication regimens. Finally, many patients in the PICU are prescribed high-risk medications, with automated safeguards built into the EHR to prevent overexposure to the medication. Pharmacists and providers should be educated on these safeguards, and EHR tools should be utilized to minimize unintended order changes.

### Specific Aims

Our primary aim was to reduce the rate of reported monthly prescriber medication errors per 1,000 medication orders by 40% between August 2022 and June 2024.

Our secondary aim was to decrease the rate of reported prescriber admission medication reconciliation errors related to the admission process for patients with CCC by 40% during the intervention period.

## METHODS

### Context

We conducted this quality improvement (QI) initiative from August 2022 through June 2024 at a large tertiary care urban children’s hospital with a 26-bed PICU. Our institution is a nearly 200-bed pediatric center located within a larger adult hospital system. All major pediatric subspecialties are represented, and there are several local chronic pediatric care facilities that admit patients directly to our PICU. Multidisciplinary rounds occur daily and include a PICU attending, fellow, hospitalist, nurse, pharmacist, and pediatric residents. Nighttime rounds occur without the presence of a PICU pharmacist. Rounds are family-centered with an opportunity for family input on the patient’s medication reconciliation.

It is our standard that medication errors found before or after reaching the patient are entered by the PICU physicians, nurses, or pharmacist into our institution’s occurrence monitoring tool, MIDAS (Medical Information Data Analysis System). This is a self-reported system that allows the reporting of adverse events, near misses, and/or unsafe conditions along with the error type that occurred.

As this system of reporting does not include a trigger tool and reversal agent, all submitted MIDAS reports were analyzed further to determine if they were true prescriber medication errors. These were further investigated if the affected patient had a CCC and the error occurred within 24 hours of a patient’s admission. The average rate of MIDAS reporting at our institution during the study period was 148 reports per month with a SD of 19.7 (**see Figure, Supplemental Digital Content 1,** https://links.lww.com/PQ9/A792). Our most recent institutional survey results utilizing the Surveys on Patient Safety Hospital Survey 2.0 indicate that 72% of those surveyed felt that witnessed patient safety errors were reported either most of the time or always.^19^ A good-catch system exists at our hospital, whereby those who utilize our error reporting system effectively are presented a “Good Catch Award” during monthly safety meetings.

This project, titled “Decreasing prescriber errors in a tertiary care pediatric intensive care unit,” was approved by the Einstein institutional review board (approval no. 125361). Informed consent needs were waived by this regulatory body. All procedures were followed in accordance with the ethical standards of the Office of Human Research Affairs and with the Helsinki Declaration of 1975.

### Planning the Intervention

**Fig. 1.**
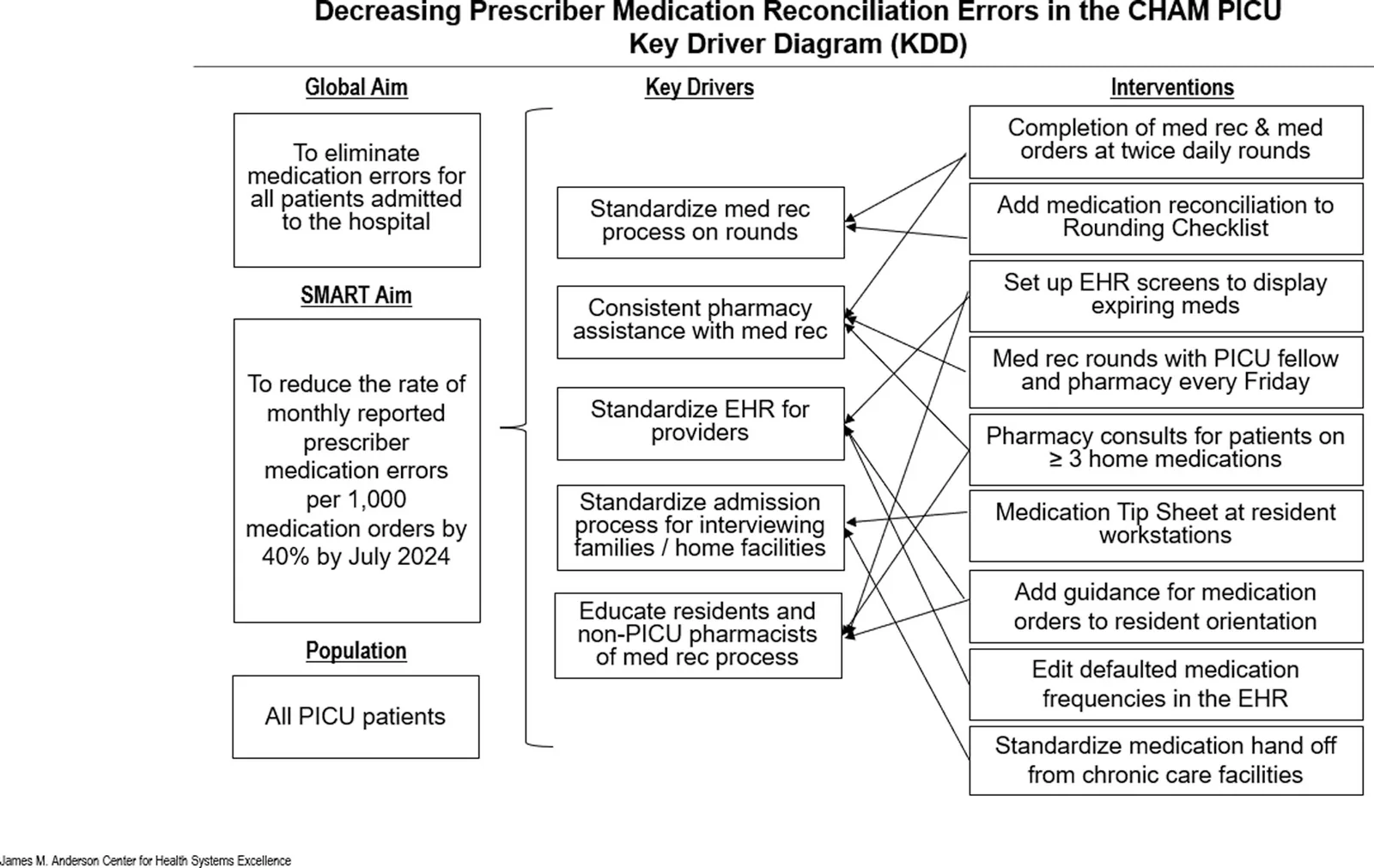
Key Driver Diagram with an aim of reducing monthly prescriber medication errors in the PICU.

Our key stakeholders included PICU attendings, fellows, hospitalists, and the PICU clinical pharmacy manager. Key drivers were identified (Fig. 1). Interventions were carried out using the model for improvement. Data on prescriber errors were collected through our error reporting system and spot checks twice weekly during bedside rounds.

### Plan-do-study-act Cycles

#### Plan-do-study-act 1: Standardization of the Medication Reconciliation Process (August 2022)

A rounding safety checklist prompted the rounding team to designate a resident to enter and modify orders and complete a medication reconciliation for the current patient before finalizing the patient’s daily plan.

#### Plan-do-study-act 2: Standardization of Resident EHR Screens to Include “Expiring Orders” Flag (October 2022)

Medications would often unintentionally expire without being renewed, leading to medication omission errors. Our EHR has an optional “Expiring Orders” flag which alerts providers that a medication will expire in 24 hours without being acknowledged. This flag was not included on the default EHR screens for residents and was often underutilized. Clicking this flag opens the orders tab in the EHR and allows the residents to either “Renew,” “Modify,” or “Let Expire.” Once the order is acknowledged it will be removed from the EHR screen.

We now include time during resident orientation to install this flag on all residents’ default EHR screens.

#### Plan-do-study-act 3: Med Rec Fridays (January 2023)

Medication errors are likely to occur over the weekend when there are fewer providers available. We implemented a 15-minute meeting every Friday between the service PICU fellows and pharmacist(s) to ensure medication orders were accurate and would not expire over the weekend. This intervention was abandoned after several weeks due to time constraints and redundancy in the process. We learned that more frequent reassessments of a patient’s current medication list were likely not needed following PDSA 1.

#### Plan-do-study-act 4: Pharmacy Education and EHR Standardization (September 2023)

Errors occurred due to discrepancies between the timing of hospital-assigned medication frequencies and patient-specific frequencies (**see Figure, Supplemental Digital Content 2,** https://links.lww.com/PQ9/A793). Prescribers were often not aware that there was a system-level misalignment when placing orders as “Q12H” or “BID” resulting in inappropriately timed medications. The Pediatric Pharmacy Department was provided education regarding this issue.

The Pediatric Pharmacy Department also standardized its EHR interfaces to include the “Expiring Orders” flag per plan-do-study-act (PDSA) 2.

#### PDSA 5: Pharmacy Medication Reconciliation Consult (January 2024)

Multiple medication errors were noted in patients with CCC due to inaccurate home medication reconciliation at the time of admission. PDSA 1-4 did not fully address this issue. Consequently, a Pharmacy Medication Reconciliation Consult (PMRC) order was built into our EHR. Ordering a PMRC prompted the pharmacist to obtain an independent home medication reconciliation for the patient. Once completed, a standardized consult note, including the home medication reconciliation information (**see Figure, Supplemental Digital Content 3,** https://links.lww.com/PQ9/A794), was placed in the patient’s chart. This process was available during daytime hours Monday through Friday and encouraged for patients with a CCC who require three or more home medications.

### Measures

The primary outcome was the rate of monthly reported prescriber medication errors per 1,000 medication orders placed. The rate of prescriber medication errors was based on submitted incident reports, whereas the total number of monthly medication orders was extracted from the EHR. On occasion, a single incident report could contain multiple prescriber medication errors, in which case each error was counted separately. Our secondary outcome was the rate of monthly reported prescriber admission medication reconciliation errors per 1,000 medication orders placed during the admission of patients with a CCC.

Spot checks were performed twice weekly during bedside rounds by one of the key stakeholders to compare prescriber errors identified on rounds with submitted safety incident reports documenting prescriber errors. All errors noted by the stakeholder during spot checks also had error reports submitted, indicating that MIDAS reports were an accurate measure of prescriber medication errors. Following spot checks, rounding hospitalists were further educated on the importance of reporting all medication errors to our error reporting system.

The stakeholder team was initially concerned about our interventions leading to increased rounding times or delays in patient care. Consensus during QI meetings and PICU faculty meetings agreed that subjective rounding times were not affected by changes enacted during this QI project.

### Evaluation and Statistical Analysis

Primary and secondary outcomes were plotted on statistical process control charts and analyzed for special cause variation, as detailed in the Healthcare Data Guide.^20^ Control charts were created using QI Macros software (KnowWare International, Inc., Denver, Colo.) for Microsoft Excel (Microsoft, Redmond, Wash.) and annotated with PDSA cycles.

## RESULTS

There was an average of 3,764 monthly medication orders placed during the six-month baseline period compared to an average of 3,254 monthly medication orders during the study period. There were no broad changes in the hospital staffing or safety policies during this time.

**Fig. 2.**
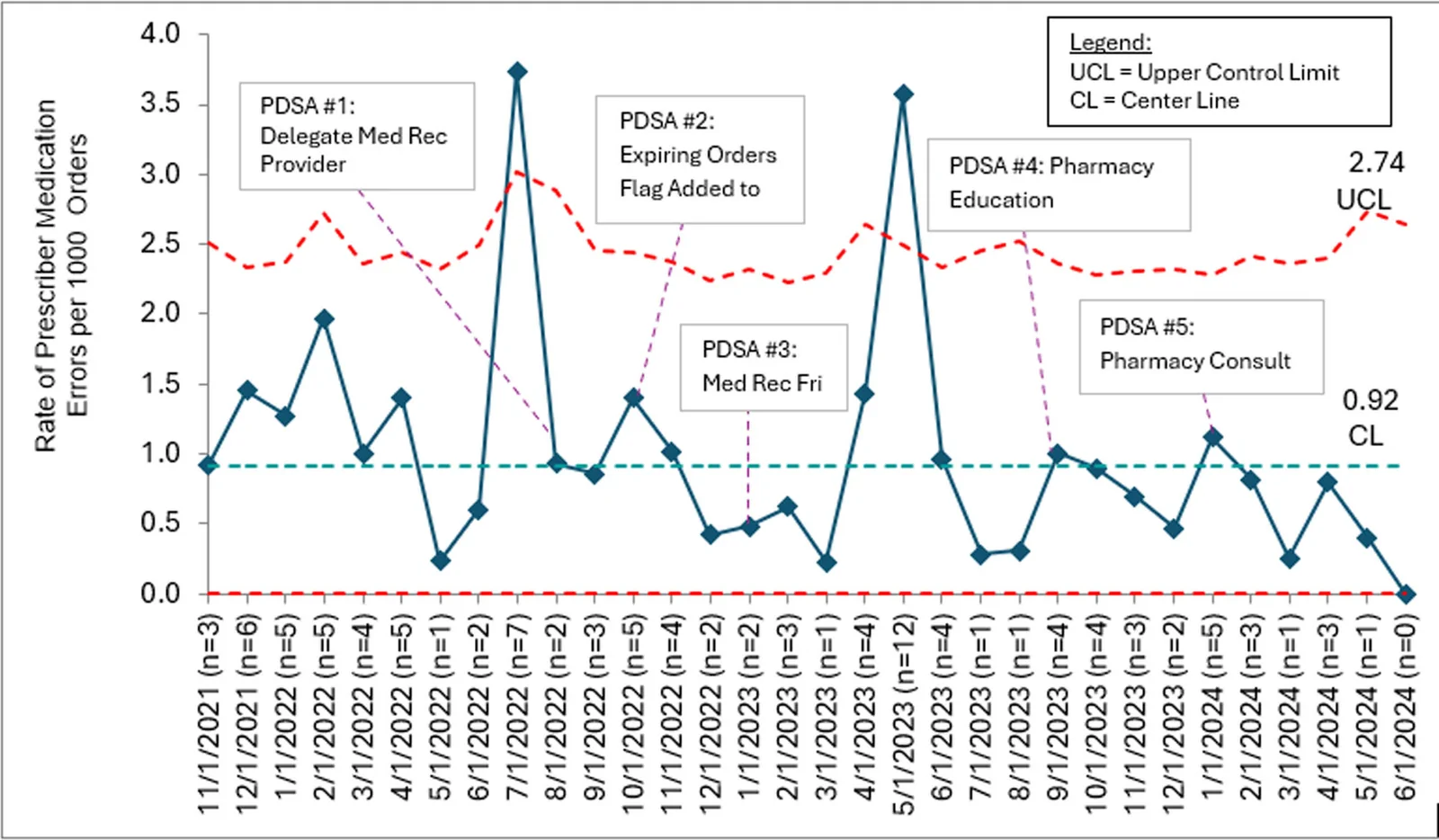
Statistical Process Chart (SPC) U-chart of monthly prescriber medication error rates. The monthly rate of reported prescriber medication errors is displayed as the number of errors per 1,000 medication orders from November 2021 to June 2024. Although variation in monthly prescriber medication error rates was observed throughout the study period, no sustained special cause variation meeting standard SPC criteria was identified. Therefore, the center line remained unchanged at 0.92 errors per 1,000 medication orders. CL, center line; U-chart, unit control chart; UCL, upper control limit.

**Fig. 3.**
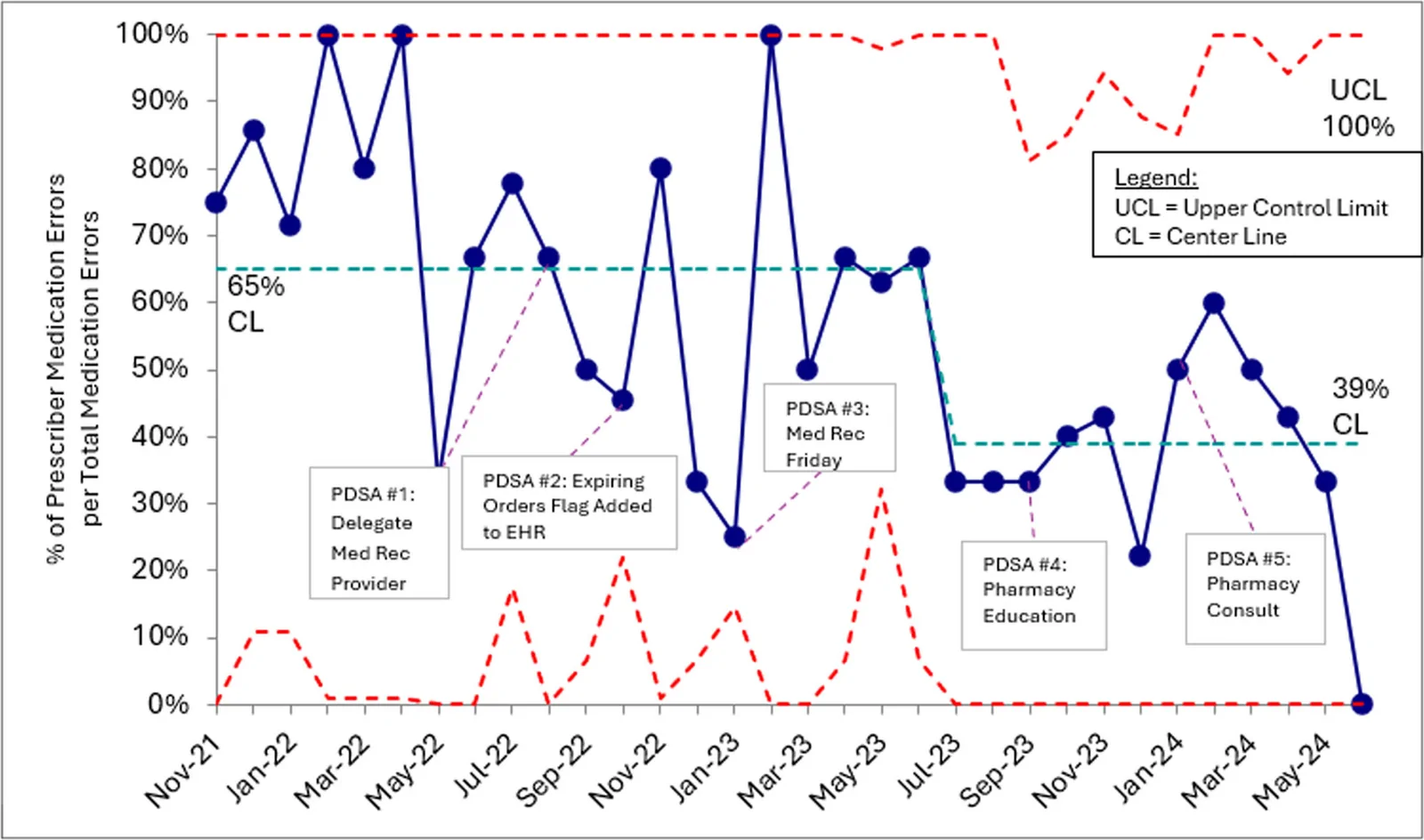
SPC P-chart of the monthly proportion of total medication error rates attributable to prescriber errors. Each point represents the percentage of all reported prescriber medication errors that were classified as prescriber medication errors during the corresponding month. Following the implementation of the pharmacy-focused interventions, the process demonstrated sustained special cause variation, resulting in a center line shift from 65% to 39%. CL, center line; P-chart, proportion control chart; UCL, upper control limit.

The rate of prescriber medication errors did not demonstrate special cause variation on statistical process control analysis, limiting the interpretation of improvement (Fig. 2). As a result, it can be concluded that there was no change in the rate of total prescriber medication errors throughout the project. However, it is important to note that the percentage of all medication error reports attributable to prescriber error decreased from 65% to 39%, a decrease of 40% from baseline, shown by a downward centerline shift starting in July 2023 (Fig. 3). Further analysis of the various subtypes of prescriber medication errors occurring within the PICU during this project can be found in **Figure (Supplemental Digital Content 4,** https://links.lww.com/PQ9/A795).

**Fig. 4.**
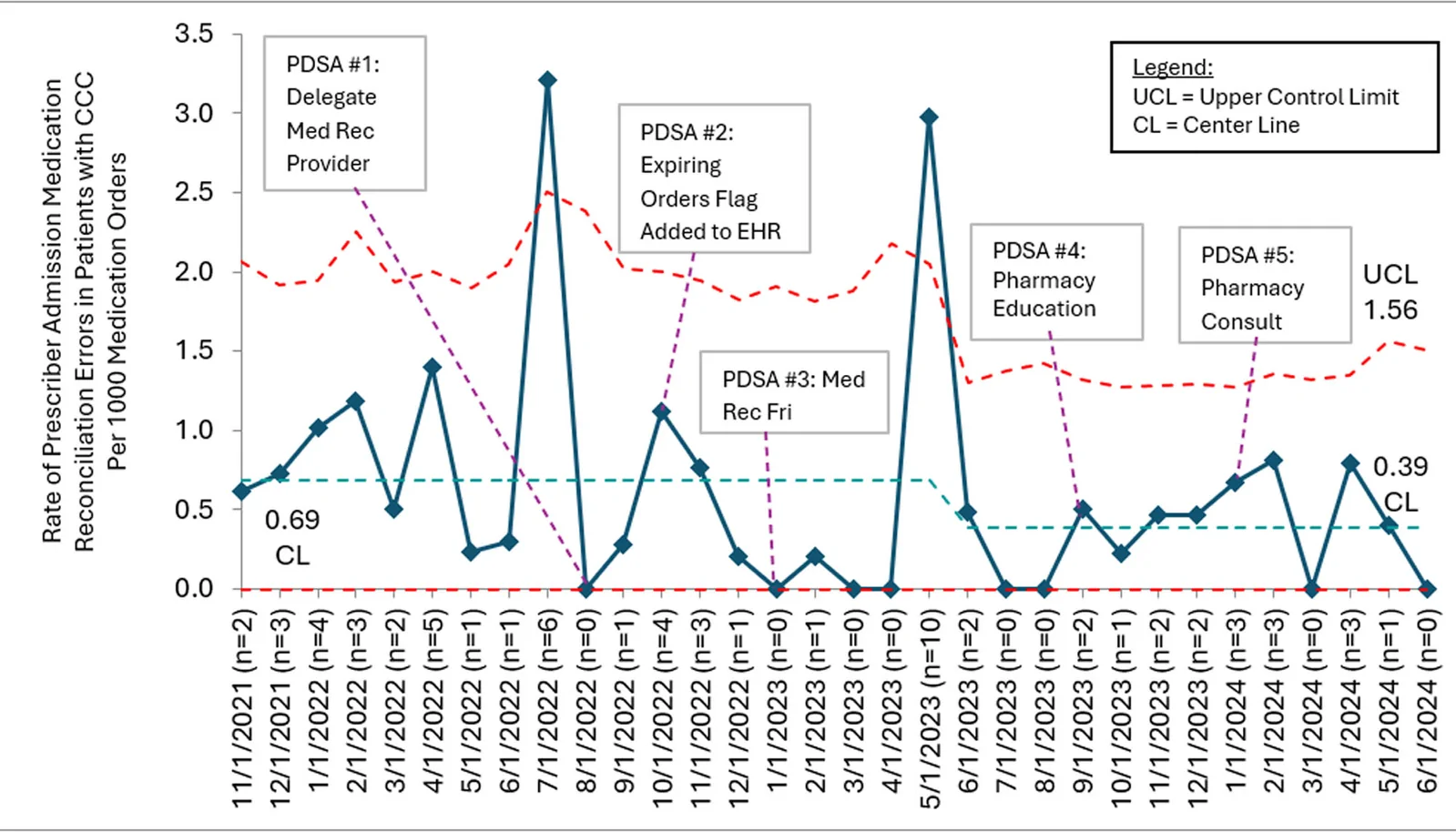
SPC U-chart of admission medication reconciliation error rates among patients with CCCs. The monthly rate of prescriber admission medication reconciliation errors among patients with CCCs is displayed as the number of errors per 1000 medication orders from November 2021 to June 2024. Following the implementation of the pharmacy-focused interventions, the process demonstrated sustained special cause variation, resulting in a center line shift from 0.69 to 0.39 errors per 1,000 medication orders. CL, center line; U-chart, unit control chart; UCL, upper control limit.

**Fig. 5.**
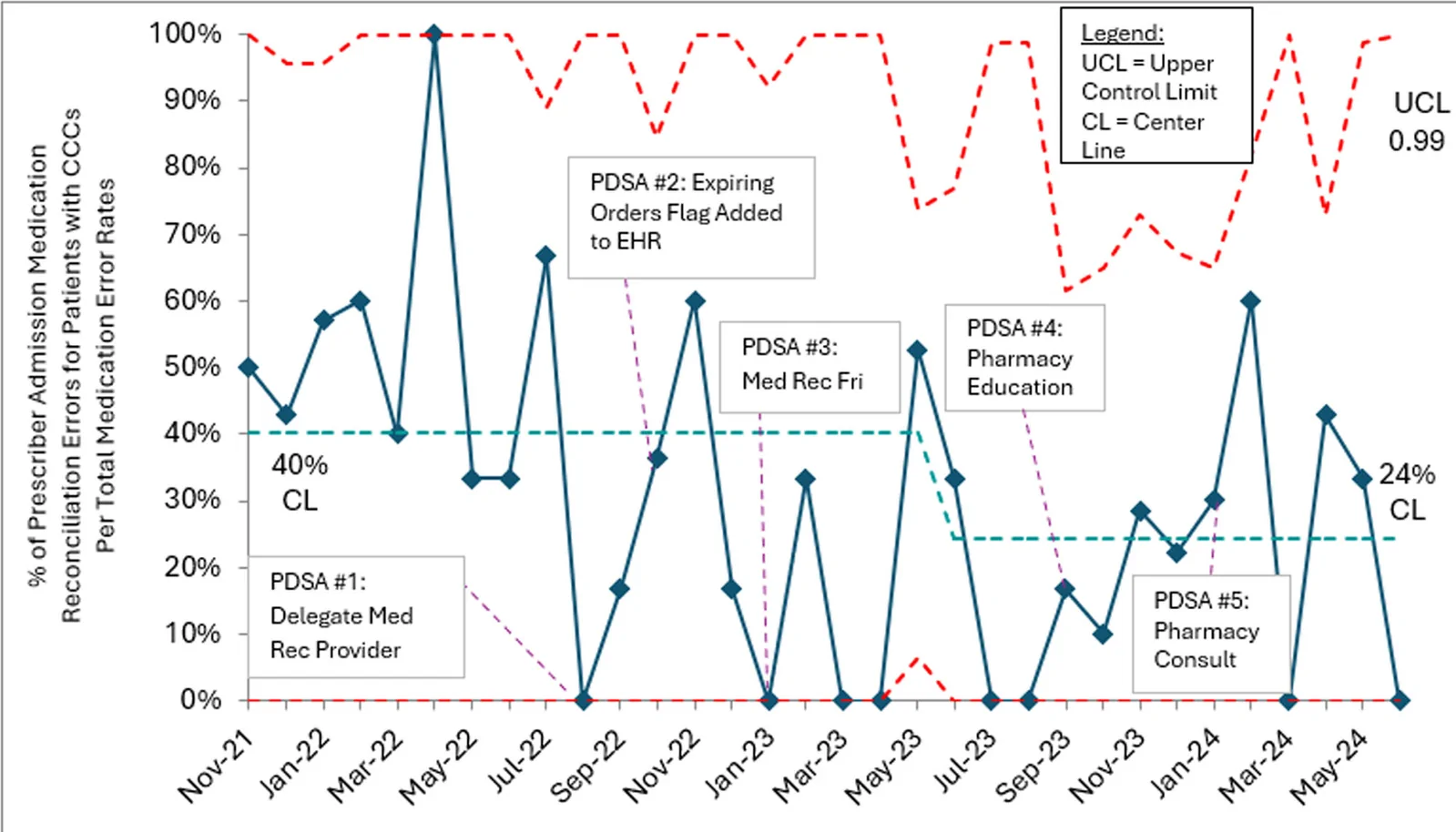
SPC P-chart of the monthly proportion of admission medication reconciliation errors among patients with CCCs. Each data point represents the monthly percentage of admission medication reconciliation errors among patients with CCCs relative to all medication errors identified during the admission process from November 2021 to June 2024. Following the implementation of pharmacy-focused interventions, the process demonstrated sustained special cause variation, resulting in a center line shift from 40% to 24%. CL, center line; P-chart, proportion control chart; U-chart, unit control chart; UCL, upper control limit.

The rate of admission medication reconciliation errors in patients with CCC per 1000 medication orders did meet special cause variation with a downward centerline shift from 0.69 to 0.39 that started in June 2023 (Fig. 4). As seen in Figure 5, the proportion of admission medication reconciliation errors for patients with CCCs per total medication errors also met special cause variation, with a downward shift of the centerline from 40% to 24%, an overall decrease of 40%. Of note, there were two astronomical data points that met special cause variation in July 2022 and May 2023. In both cases, there were multiple patients with CCC admitted to the PICU with extensive outpatient medication lists that were not accurately reconciled on admission.

Data were obtained from the 36 completed PMRCs, with only 4 PMRC orders placed without a consult completed. The delay from PMRC order placement to note completion ranged from 0 to 2 days, with an average of 0.3 days. Further data gained from PMRCs are present in Table 1.

**Table 1.** Data Collected from Completed PMRC

PMRCs (N = 36)	
Admitted from	
Home, N (%)	21 (58)
Long-term care facility, N (%)	15 (42)
Medication reconciliation completed with	
Patient and/or CAREGIVER, N (%)	18 (50)
Provider, N (%)	6 (16.7)
Long-term care facility, N (%)	17 (47.2)
No. home medications Reconciled, N	335 (9.3/patient)
Neurologic medications, N	103
Gastrointestinal medications, N	104
Respiratory medications, N	68
Renal medications, N	5
Infectious disease medications, N	4
Miscellaneous medications, N	51
Error frequencies	
Patients with prescriber error present, N (%)	19 (53)
No. errors noted	21
Type of med error	
Wrong dose, N (%)	7 (33)
Wrong frequency, N (%)	1 (5)
Inappropriate timing of meds, N (%)	9 (43)
Omitted med, N (%)	4 (19)

PMRCs were completed 36 times. Patients were on an average of 9.3 home medications. PMRCs discovered a prescriber error in 53% of cases. The majority of errors were due to inappropriate medication timing (43%) or incorrect doses (33%).

## DISCUSSION

In this QI initiative, our aim was to reduce the rate of reported prescriber medication errors in critically ill pediatric patients per 1,000 medication orders. Although we did not decrease the overall rate of prescriber errors, the proportion of medication errors related to prescriber errors, especially during the admission of patients with a CCC, did decrease over the course of the study period. This was particularly seen following the implementation of a Pharmacy Medication Reconciliation Consult process. Formally incorporating a process to confirm that ordered medications match a planned medication regimen can help to minimize prescriber errors. Using the EHR to minimize or capture latent errors, such as flagging expiring medications, can further reduce prescriber errors.

As we began to adopt our PDSA cycles, it became clear that patients with a CCC required further interventions and a larger focus in our study. It is important to highlight patients with a CCC as they are a growing patient population in the PICU. The analysis of those patients with a CCC who received a PMRC showed that 53% of these patients had errors related to admission medication reconciliation, which is comparable to prior studies,^7,8,10^ with most errors related to incorrect medication dosing or timing. Compared to a prior study of 23 medically complex children,^8^ patients receiving a PMRC at our institution were on more home medications (9.3 versus 5.3 per patient) with fewer errors related to the omission of medications (19% versus 45%). A more recent QI study^9^ analyzing medication reconciliation errors during transitions of care within a public hospital also noted higher rates of omission errors than seen during our study (47.2%–58.3% versus 19%). Patients with a CCC often have complex home medication regimens and likely warrant pharmacy involvement in their admission medication reconciliation process. PMRCs have continued to be regularly placed following the completion of our PDSA cycles, with an additional 44 consults completed in the 18 months following our interventions.

Our efforts were part of a broader goal across our institution to expand and promote our error reporting system to assist in identifying latent safety issues. Our large volume of reports, with a low monthly deviation in reported rates during the study period, implies stability and appropriate capture of self-reported safety events (**Supplemental Digital Content 1,** https://links.lww.com/PQ9/A792). We believe the increased reporting of errors when compared to the period before the initiation of our study is suggestive of more accurate data collection as well. Despite its subjective nature, our twice-weekly spot checks found similar errors to those submitted to the error reporting software.

A significant limitation in our analysis is our reliance on a subjective and voluntary error reporting system, which may be affected by the reporting individual’s familiarity with the reporting process or willingness to report errors. Westbrook et al found in their multicenter study comparing submitted incident reports to over 11,000 errors found through manual audits a remarkably low rate of only 3.2 of 1,000 medication errors with an associated incident report.^21^ Incident reporting systems capture only a fraction of medication errors compared with structured detection methodologies such as pediatric trigger tools. Stockwell et al and Kirkendall et al demonstrated substantially higher adverse event detection using trigger-based methodologies than incident reporting alone.^22,23^ Future work should incorporate trigger tools and reversal-agent surveillance to provide a more comprehensive assessment of medication safety.

We were unable to clarify the proportion of patients eligible to receive a PMRC who had a formal consult placed or the proportion of expiring orders that were addressed. We are thus unable to determine if PDSA 2 or 5 led directly to improved medication reconciliation or if our improvements were due to an extraneous factor. Additionally, although the PMRC allowed prescriber errors to be caught early, initial errors could not be prevented from reaching the patient. In our study, there was one adverse drug event by omission, as a patient with a CCC had a seizure in the setting of not receiving a home antiepileptic drug.

Future directions include adjusting the default ordering frequencies to minimize medication errors related to incorrect timing. Forty-three percent of medication reconciliation errors for patients with a CCC were related to inaccurate medication timing. All key stakeholders have approved removing the “BID” frequency as an option for inpatient medications pending the integration of this change into the system’s EHR. We hope to streamline the admission process for patients with a CCC who reside in long-term care facility by developing a standardized interinstitutional medication reconciliation hand-off. Finally, we hope to expand the PMRC to be available outside of daytime hours and have broader patient eligibility.

## CONCLUSIONS

A multidisciplinary QI approach was associated with improvement in medication reconciliation processes for children with a CCC, as seen by a decrease in admission medication reconciliation errors. Pharmacy-supported medication reconciliation and standardized workflows were associated with fewer admission medication reconciliation errors in this high-risk population. Future directions include expanding our pharmacy consultation to assist in complex medication reconciliation outside of the PICU.

## Supplementary Material


