# Supplementary material for: Decreasing Prescriber Errors in a Tertiary Care Pediatric Intensive Care Unit via QI Methodology
Source: Pediatr Qual Saf. 2026 Sep 22;11(5):e909. doi: 10.1097/pq9.0000000000000909 (PMC13596933; doi:10.1097/pq9.0000000000000909)
Supplement: Supplementary file 1 [file pqs-11-e909-s001.pdf]

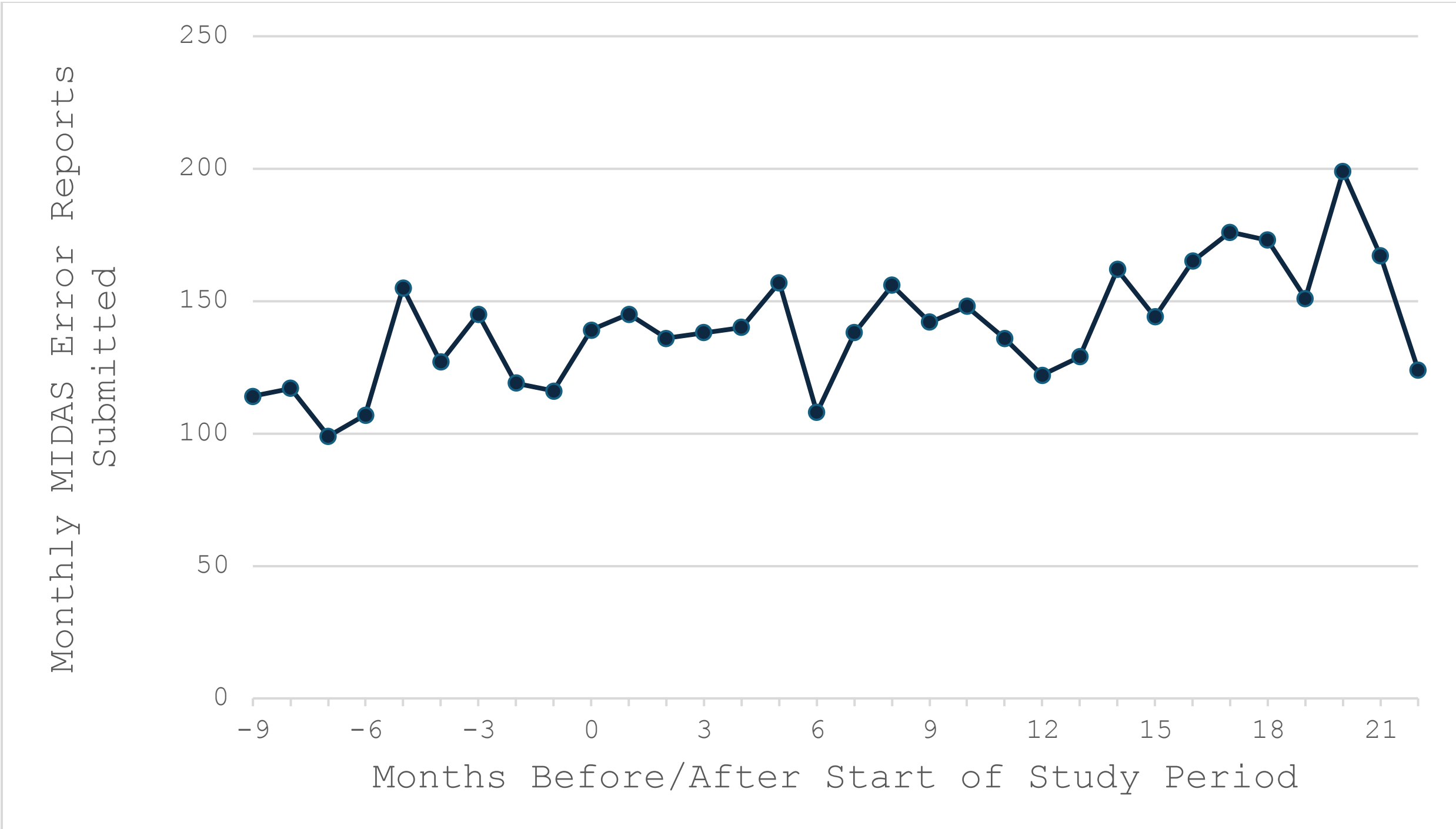

Figure SDC 1. Analysis of submitted MIDAS error reports throughout our institution beginning 9 months prior to study intervention period. Throughout the study period, there was an average of 148 monthly submitted reports with a standard deviation of 19.7 reports per month. There was an average of 122 monthly submitted reports prior to our study period.
