# Supplementary material for: Decreasing Prescriber Errors in a Tertiary Care Pediatric Intensive Care Unit via QI Methodology
Source: Pediatr Qual Saf. 2026 Sep 22;11(5):e909. doi: 10.1097/pq9.0000000000000909 (PMC13596933; doi:10.1097/pq9.0000000000000909)
Supplement: Supplementary file 2 [file pqs-11-e909-s002.pdf]

Decreasing Medication Reconciliation Errors in a Tertiary Care Pediatric Intensive Care Unit
Timothy Brandt

SDC, Figure 3

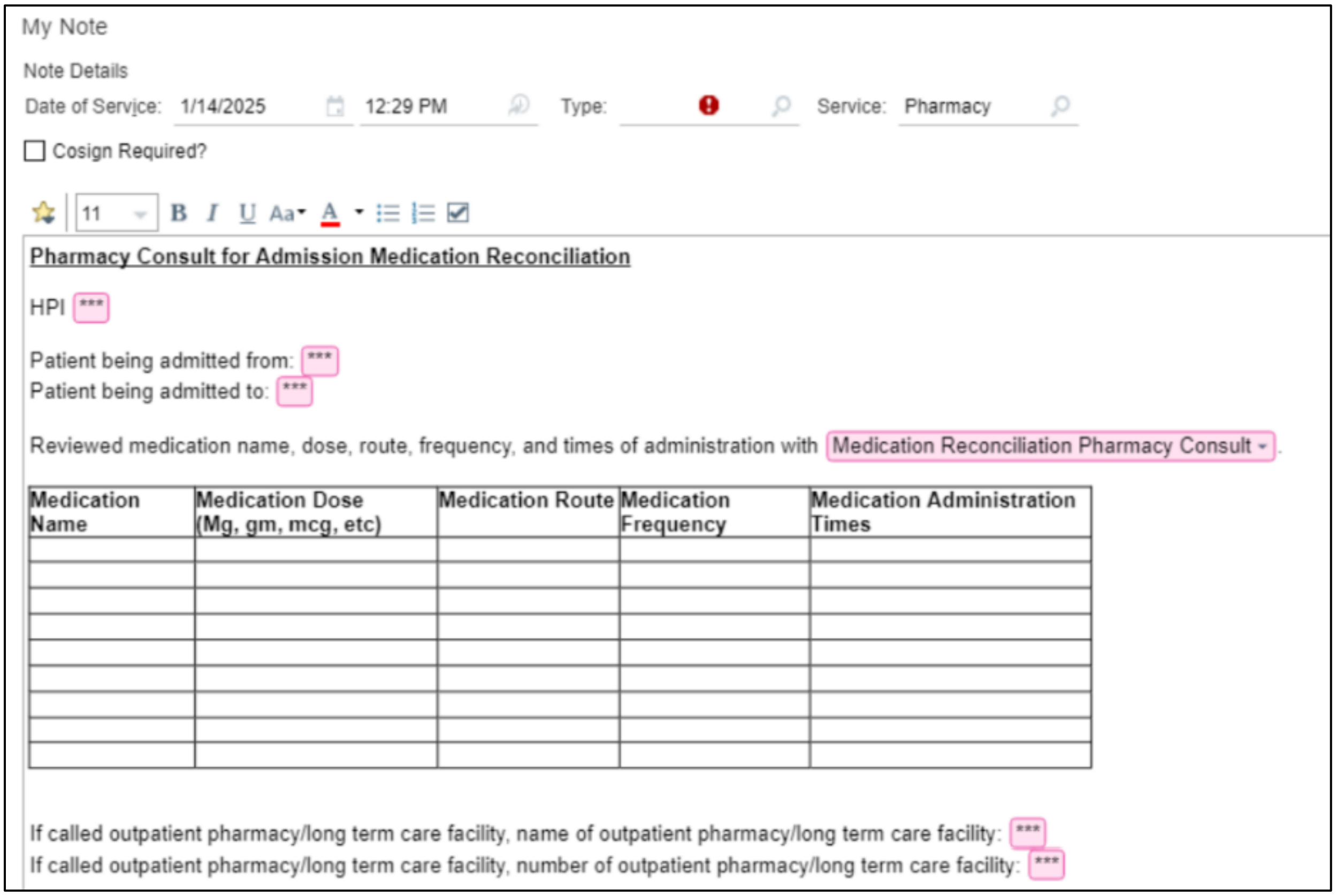
My Note

Note Details

Date of Service: 1/14/2025 12:29 PM Type: Service: Pharmacy

☐ Cosign Required?

11 B I U Aa A

Pharmacy Consult for Admission Medication Reconciliation

HPI ***

Patient being admitted from: ***
Patient being admitted to: ***

Reviewed medication name, dose, route, frequency, and times of administration with Medication Reconciliation Pharmacy Consult.

Medication Name	Medication Dose (Mg, gm, mcg, etc)	Medication Route	Medication Frequency	Medication Administration Times

If called outpatient pharmacy/long term care facility, name of outpatient pharmacy/long term care facility: ***
If called outpatient pharmacy/long term care facility, number of outpatient pharmacy/long term care facility: ***

Figure SDC 3. Template for Pharmacy Medication Reconciliation Consult note. Note includes medication name, dosage, route, frequency, and timing of administration. This note is entered into the EHR and viewable throughout admission and during any subsequent admissions.
