# Supplementary material for: Decreasing Prescriber Errors in a Tertiary Care Pediatric Intensive Care Unit via QI Methodology
Source: Pediatr Qual Saf. 2026 Sep 22;11(5):e909. doi: 10.1097/pq9.0000000000000909 (PMC13596933; doi:10.1097/pq9.0000000000000909)
Supplement: Supplementary file 4 [file pqs-11-e909-s004.pdf]

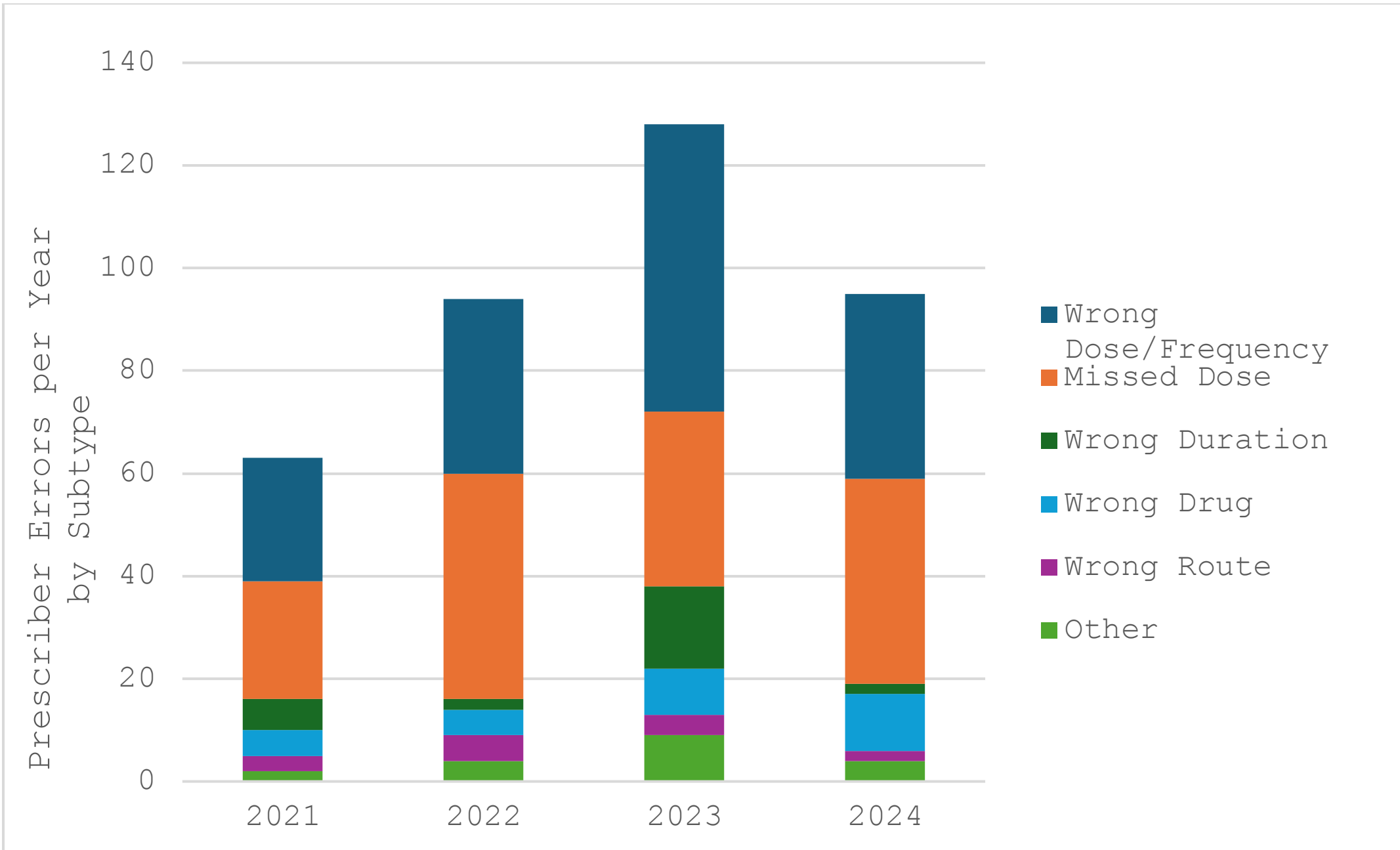

Figure SDC 4. Prescriber Errors in PICU per year divided into subtypes of prescriber errors. Of note, taxonomy classification of error subtypes figure was automated via occurrence monitoring software.
